# Investigation of the Usefulness of Implants With Locking Mechanisms for Diffuse Idiopathic Skeletal Hyperostosis (DISH)-Induced Thoracic and Lumbar Fractures in Patients Operated in the Prone Position

**DOI:** 10.7759/cureus.67071

**Published:** 2024-08-17

**Authors:** Seiya Watanabe, Kazuo Nakanishi, Kazuya Uchino, Hideaki Iba, Yoshihisa Sugimoto, Shigeru Mitani

**Affiliations:** 1 Orthopaedics, Kawasaki Medical School, Kurashiki, JPN

**Keywords:** implants with locking mechanisms, local kyphosis, posterior fusion, prone position, dish

## Abstract

Background

Diffuse idiopathic skeletal hyperostosis (DISH) is a disease that causes bone growth in the spine and musculoskeletal system, and even minor trauma can cause fractures that often require surgery. DISH-induced fractures show a tendency for bone loss when operated in the prone position, which can lead to poor fusion and implant failure; therefore, surgery in the lateral recumbent position is often recommended. However, inserting a pedicle screw (PS) in the lateral recumbent position is technically difficult. This study examined the effectiveness of the repair and fixation of thoracic and lumbar spine fractures using implants with locking mechanisms in the prone position in patients with DISH.

Methods

We retrospectively analyzed the data from 11 patients (six males and five females; mean age: 87 years) who underwent surgery for thoracic and lumbar fractures caused by DISH between December 2023 and June 2024. Surgery was performed in the prone position using PSs or transdiscal screws (TSDs) for DISH. Ennovate® implants manufactured by B-BRAUN were used. The fixed range was three above-three below for PSs and two above-two below for TSDs. The evaluation parameters were the height/level of injury, operative time, blood loss, local kyphosis angle, anterior wall height ratio, and complications. The local kyphosis angle was measured as the angle between the upper and lower endplates of the fractured vertebrae. The ratio of the anterior wall height was evaluated.

Results

The average operative time was 87 min (52-172 min), and the average blood loss was 40ml (10-140 ml). The preoperative and postoperative local kyphosis angle was −8.7° and −2.4°, respectively, and the average local kyphosis angle improvement was 6.3° (0.1-14°). The preoperative and postoperative anterior wall height ratio was 132% and 110%, respectively, and the average anterior wall height ratio improvement was 22% (2-82%). No complications, such as screw deviation, implant loosening, loss of correction, or skin problems, were observed.

Conclusion

This study demonstrated that DISH-induced thoracic and lumbar spine fractures could be repaired and fixed using implants with locking mechanisms in the prone position. The prone position is familiar to spine surgeons and is considered safe. Additionally, screw migration may occur due to decreased bone density in the vertebral bodies with DISH; in such cases, it would be better to fix the screw without forcing it to be repositioned.

## Introduction

Diffuse idiopathic skeletal hyperostosis (DISH) is a disease that causes bone growth in the musculoskeletal system, including the spine [1]. When DISH vertebrae are exposed to external forces, the stress tends to concentrate, and fractures occur even with minor trauma such as falls [2]. DISH-induced fractures are highly unstable and may cause delayed neuropathy, which may require surgery. When a patient with DISH is operated on in the prone position, the anterior part of the vertebral body opens and bone loss occurs. Fixation of residual fractures can lead to poor fusion and implant failure [3]. Surgeries performed in the lateral recumbent position to prevent the anterior opening of the vertebral body have been reported [4]. Flexing the spine in the lateral recumbent position allows the fracture to be repositioned and immobilized in an appropriate alignment. However, pedicle screw (PS) placement in the lateral recumbent position is a complicated procedure that is often difficult to observe using fluoroscopy and requires experience. If the PS deviates, major complications such as spinal cord injury may occur. We needed to devise a way to insert the PS safely and treat the fracture site as if it were in the lateral recumbent position. Therefore, we investigated the effectiveness of treating DISH-induced thoracolumbar fractures using implants with locking mechanisms in the prone position of the patients, which is a position familiar to most spine surgeons.

## Materials and methods

**Figure 1:**
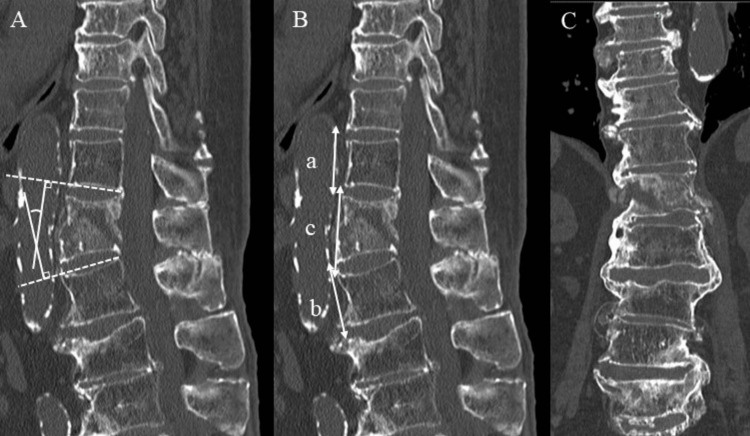
Image evaluation in CT A. Local kyphosis angle (°); B. Ratio of the anterior wall height (%); C. Plain CT coronal section The formula for the ratio of the anterior wall height is shown. A value closer to 100% indicates that the vertebral height of the affected vertebrae is reduced closer to its original height. The ratios of the anterior wall height of the cephalocaudal adjacent vertebral body to the fractured vertebrae with the adjacent craniocaudal disc height (a & b) and the anterior wall height of the fractured vertebral body with the adjacent craniocaudal disc height (c) are measured using this image.

The study was approved by the Ethics Review Committee of Kawasaki Medical School (approval number: 6481-00). We retrospectively analyzed the data from 11 patients (six males and five females; mean age: 87 years) who underwent surgery for DISH-induced thoracic and lumbar fractures between December 2022 and June 2023. The mean follow-up is eight months (4-13 months). Causes of injury were attributed to falls in nine cases and traffic-accident trauma in two cases. The surgery was performed in the prone position with posterior fixation achieved using a PS and a transdiscal screw (TSD) for DISH [5]. Ennovate® implants manufactured by B-BRAUN (Melsungen, Germany) were used. The fixed range was three above-three below for PSs and two above-two below for TSDs. The evaluation parameters were the level of injury, Thoracolumbar Injury Classification System (TLICS), Thoracolumbar AO Spine Injury Score (TLAOSIS), operative time, blood loss, local kyphosis angle, anterior wall height ratio, bone fusion, and complications. The local kyphosis angle was measured as the angle between the upper and lower endplates of the fractured vertebra. The anterior wall height ratio was evaluated using the method described by Ikuma et al. [5] (Figures 1A, 1B). The diagnosis of DISH was ossification or calcification of four or more contiguous vertebrae evaluated by plain computed tomography (CT) sagittal and coronal sections [1] (Figure 1C). The definition of bone fusion was defined as fusion in both sagittal and coronal sections of CT.

Reduction technique

**Figure 2:**
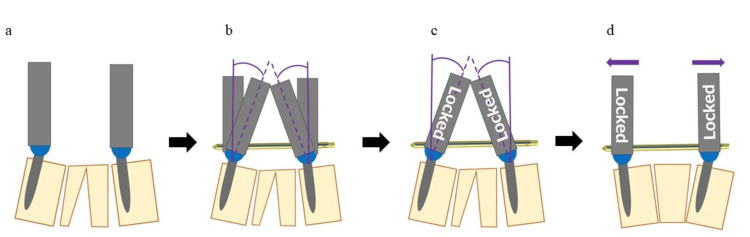
Reduction technique a. Insertion of screws with locking mechanisms into the upper and lower vertebrae of the fractured vertebra; b. Tilting the extender to the required angle to crimp the forward opening after inserting the rod; c. Poly-locking and monoaxializing the screw heads; d. Straightening the extender until it is parallel and fixing it with set screws This figure was created by one of the authors, Kazuya Uchino.

General anesthesia was induced with the patient in the prone position lying on a pillow on the Jackson Table (Howell Medical, China) to keep the fracture as closed as possible. A screw with a locking mechanism was inserted into the screw head under fluoroscopy. After inserting the rod, the extender was tilted to an appropriate angle for crimping the forward extension. The screw head was locked and monoaxialized. The extender was corrected until it was parallel (Figures 2A-2D). Finally, the extender was fixed with set screws.

Statistical analysis

Preoperative and postoperative local kyphosis angles and ratios of the anterior wall height were compared using the Wilcoxon rank sum test; P-values <0.05 were considered statistically significant.

## Results

In this study, 11 cases were evaluated (Table 1).

**Table 1:** Details of the 11 cases included in the study TLICS: Thoracolumbar Injury Classification System; TLAOSIS: Thoracolumbar AO Spine Injury Score

Case	Age (yr.)	Sex	Level	TLICS	TLAOSIS	OP time (min)	Blood loss (ml)	Local kyphosis (°)			Ratio of anterior wall height (%)			Bone fusion	Complication
								Pre	Post	Δ	Pre	Post	Δ		None
1	89	M	T11	5	6	125	100	-5.0	-4.1	0.9	152	140	12	+	None
2	86	F	L2	5	7	172	100	-39.5	-25.5	10.0	234	152	82	-	None
3	84	F	T8	5	6	64	10	-2.0	-0.4	1.6	124	98	26	+	None
4	76	F	T12	4	5	69	10	17.0	28.0	12.0	135	101	34	+	None
5	87	M	L3	5	6	74	10	-11.0	-7.0	4.0	136	120	16	+	None
6	80	M	L1	4	6	78	10	-12.7	-3.6	9.1	116	104	12	+	None
7	95	F	L1	4	5	124	140	-21.0	-10.8	10.2	132	107	25	+	None
8	94	F	T11	5	6	64	10	1.2	11.7	10.5	126	106	20	+	None
9	91	F	T9	3	4	72	35	-5.5	-4.4	1.1	116	114	2	+	None
10	96	M	T11	8	10	67	20	-2.3	-2.2	0.1	115	108	7	-	None
11	78	M	T10	5	5	52	10	-15.1	-8.2	6.9	93	107	6	+	None

**Figure 3:**
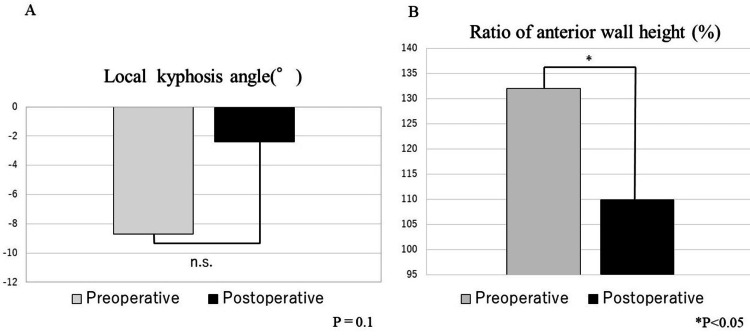
Pre- and postoperative evaluation of the local kyphosis angle and anterior wall height ratio A. The local kyphosis angle has improved from −8.7° preoperatively to −2.4° postoperatively, without a statistically significant difference; B. The average preoperative and postoperative anterior wall height ratio is 132% and 110%, respectively; the corrected anterior wall height ratio has improved significantly by 22% (2–82%). Pre- and postoperative local angles and pre and postoperative ratio of anterior wall height were compared using Wilcoxon's rank sum test. P<0.05 was considered statistically significant.

The average operative time was 87 min (52-172 min), average blood loss 40 ml (10-140 ml), average preoperative local kyphosis angle −8.7°, and average postoperative local kyphosis angle −2.4°. Although the average local kyphosis angle improvement was 6.3° (0.1-14°), the difference was not significant (P=0.1). The average preoperative and postoperative anterior wall height ratio was 132% and 110%, respectively. The average anterior wall height ratio improvement was 22% (2-82%), with a statistically significant difference (P=0.04) (Figures 3A, 3B). The fusion rate is 81.8% (9/11 cases).

No complications, such as screw deviation, implant loosening, loss of correction, or screw head-associated skin problems, were reported.

Case presentation

**Figure 4:**
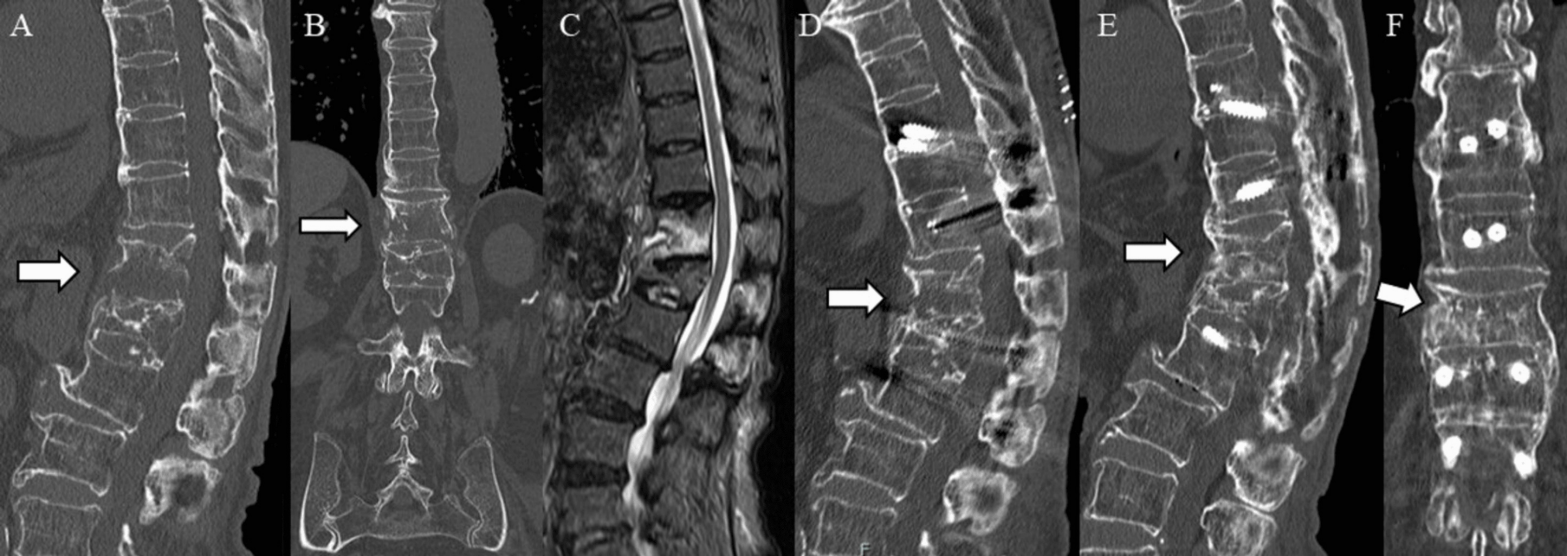
Pre- and postoperative images A. Preoperative plain computed tomography in the sagittal section (local kyphosis angle, 17°; anterior wall height ratio, 135%); B. Preoperative plain computed tomography in the coronal section; C. Preoperative magnetic resonance imaging (MRI) in the sagittal section; D. Postoperative plain computed tomography in the sagittal section (local kyphosis angle, 28°; postoperative anterior wall height ratio, 101%); E. Plain CT sagittal section at one year postoperatively. Good bone fusion; F. Plain CT coronal section at one year postoperatively. Good bone fusion.

A 76-year-old woman fell and developed back pain (Case #4; Table 1, Figure 4).

Plain CT revealed a burst fracture of the 12th thoracic vertebra associated with DISH. General anesthesia was induced, and posterior fixation was performed in the prone position. The fixed range was two above-two below. The local kyphosis angle improved by 11° (from 17° to 28°). The anterior wall height ratio improved by 34% (from 135% to 101%). The operative time was 69 min and blood loss was 10 g. Three weeks postoperatively, the patient was able to walk and was discharged. CT at one year postoperatively showed good bony fusion.

## Discussion

DISH is a disease that causes bone growth in the musculoskeletal system, including the spine [1]. DISH-induced fractures are highly unstable and may cause delayed neuropathy, which may require surgery. Thoracic and lumbar fractures due to DISH are unstable, so if posterior fixation is performed, the range of fixation should be three vertebrae up and three vertebrae down [6,7]. In contrast, thoracolumbar junction fracture is controversial in its range of fixation. There are also reports that even without DISH, three vertebrae up and three vertebrae down is essential for fixation of the thoracolumbar junction fracture due to its anatomical characteristic [8]. Short segment fixation for thoracolumbar fractures may also result in implant failure [9]. On the other hand, some reports indicate that there is no difference in postoperative results between long and short fixation [10-12]. The fracture in this study was due to DISH and required long fixation; the TSD technique was used in this study because of its increased fixation strength [5].

We investigated the usefulness of implants with locking mechanisms for DISH-induced thoracic and lumbar fractures in patients operated in the prone position. If surgery is performed in the prone position, unexpected fixation in the extended position is possible [4]. However, in this study, the average improvement in the local angle of the fractured vertebra was 6.3° and that in the anterior wall height ratio was 22%. No complications, such as screw deviation or implant loosening, were observed.

The local angle can be improved with a locking implant, even if the surgery is performed in the prone position. Many techniques have been reported for posterior fixation in DISH. Ikuma et al. reported a TSD technique that is stronger than PSs for DISH vertebrae [5]. Inoue et al. reported a cement-augmented PS that increases the fixation force and reduces the extent of fixation [13]. These techniques are intended to increase the fixation force of the screw and do not mention the reduction.

While there are methods for repairing fractures that use ligamentotaxis to restore the vertebral body, our method aims to do the opposite: to compress the fracture [14].

Spinal surgeons prefer performing posterior fusion with the patient in the prone position. However, prone positioning under general anesthesia can further exacerbate dislocation because the erector spinae muscles are relaxed [15]. Therefore, there are various reports of anterior openings of fractures being repaired in positions other than the prone position. Reinhold et al. reported a method of performing the repair in the seated position, known as the inclined-upright sitting position [16]. Furthermore, there are reports of PS insertion in the lateral recumbent position [4]. Flexion in the lateral recumbent position allows fracture reduction and fixation in an appropriate alignment. However, the PS insertion in the lateral recumbent position is difficult and requires experience. Most reports of PS insertion in the lateral recumbent position have been performed under navigation guidance [17-20]. Many reports have shown that the rate of PS deviation in the lateral position does not differ significantly from that in the prone position. However, they have all been performed under navigation guidance, and accessibility to these facilities is limited. Although we have experience with PS in the lateral recumbent position, fluoroscopic visibility is worse than that in the prone position and places a greater burden on the surgeon. Moreover, the extender may interfere with the bed and deviate outward when the lower screw is inserted in obese patients [17]. Okuda et al. reported that the lower screw deviated outward by 5° less than the upper screw in terms of inward inclination [17]. Therefore, we considered using implants that cause less stress to the surgeon and allow the fracture to be repaired as much as possible. Implants with locking mechanisms can be repositioned in the prone position, reducing the stress on the surgeon and making surgery safer. However, DISH vertebrae often have significantly reduced bone density within the vertebral body [2]. This causes the screws to move within the vertebrae in some cases before the fracture is repaired, resulting in inadequate repair. In such cases, fixation must be performed with the alignment as it is, without forcing the patient to reposition. A Japanese multicenter study reported that DISH fractures cause aortic aneurysms in approximately 0.7% of the cases [21]. Shoji et al. reported a pseudoaneurysm with a postoperative local kyphosis angle of 30° and at a distance of 30 mm after a DISH-induced fracture [22]. The ideal kyphosis angle at the thoracolumbar spine junction is less than 4 degrees [8]. However, for patients with pre-fracture kyphotic deformity, we consider it appropriate to return the patient to original alignment. Or we are trying to improve the fracture up to the preoperative CT (supine position).

DISH-related vertebral fractures tend to occur in elderly patients with a high prevalence of comorbidities, leading to perioperative complications and death. Some institutions have reported a mortality rate of 6.1-20.0% for vertebral fractures associated with DISH [23,24]. Although the mean age of the patients in this study was 87 years, which was older than that reported in other studies, no postoperative complications or perioperative deaths were reported [24,25].

The limitations of this study are its small sample size and short follow-up. The number of cases in this study is a limitation of the lack of Power analysis. It is also the lack of pre- and postoperative evaluation by ODI and other means. Future studies should include a greater number of cases and report long-term results.

## Conclusions

We evaluated DISH-induced thoracolumbar fractures operated in the prone position using implants with locking mechanisms. Operating in the prone position is familiar to most spine surgeons and is a safe technique. DISH vertebrae have reduced bone density, and the screws may move within the vertebrae when implants with locking mechanisms are used; hence, in such cases, the fracture is fixed in its original alignment.
